# Supplementary material for: The cellular architecture of memory modules in Drosophila supports stochastic input integration
Source: eLife. 2023 Mar 14;12:e77578. doi: 10.7554/eLife.77578 (PMC10069864; doi:10.7554/eLife.77578)
Supplement: Transparent reporting form [file elife-77578-transrepform1.pdf]

eLIFE
1st Floor
24 Hills Road
Cambridge CB2 1JP, UK
P 01223 855340
W elifesciences.org
T @elife

eLife's transparent reporting form

We encourage authors to provide detailed information *within their submission* to facilitate the interpretation and replication of experiments. Authors can upload supporting documentation to indicate the use of appropriate reporting guidelines for health-related research (see EQUATOR Network), life science research (see the BioSharing Information Resource), or the ARRIVE guidelines for reporting work involving animal research. Where applicable, authors should refer to any relevant reporting standards documents in this form.

If you have any questions, please consult our Journal Policies and/or contact us: editorial@elifesciences.org.

Sample-size estimation

- You should state whether an appropriate sample size was computed when the study was being designed
- You should state the statistical method of sample size computation and any required assumptions
- If no explicit power analysis was used, you should describe how you decided what sample (replicate) size (number) to use

Please outline where this information can be found within the submission (e.g., sections or figure legends), or explain why this information doesn't apply to your submission:

Electrophysiological analysis of MBON-alpha3:
We initially recorded from 5 neurons (3 with detailed information) using mCD8-GFP as a marker for our patch clamp analysis. We now added 4 more independent recordings using cytosolic EGFP as a marker and could verify all our initial results. This sample size is in line with other patch clamp recordings as these are technically very challenging. The information is presented in the results section associated with Figure 1 (including tables and supplemental information).

Computational simulations:
For the computational simulations, we either simulated all synaptic sections, all KCs, or performed trials of 1000 different combinations. 1000 trials allow for a robust evaluation of all parameters using standard statistical approaches. This information is presented in the legends to figure 3, figure 4 and figure 5 and within the methods section.

Replicates

- You should report how often each experiment was performed
- You should include a definition of biological versus technical replication
- The data obtained should be provided and sufficient information should be provided to indicate the number of independent biological and/or technical replicates
- If you encountered any outliers, you should describe how these were handled
- Criteria for exclusion/inclusion of data should be clearly stated
- High-throughput sequence data should be uploaded before submission, with a private link for reviewers provided (these are available from both GEO and ArrayExpress)

eLife Sciences Publications, Ltd is a limited liability non-profit non-stock corporation incorporated in the State of Delaware, USA, with company number 5030732, and is registered in the UK with company number FC030576 and branch number BR015634 at the address 1st Floor, 24 Hills Road, Cambridge CB2 1JP | August 2014

Please outline where this information can be found within the submission (e.g., sections or figure legends), or explain why this information doesn't apply to your submission:

Electrophysiology:
We initially recorded from 5 neurons (3 with detailed information) using mCD8-GFP as a marker for our patch clamp analysis. We now added 4 more independent recordings using cytosolic EGFP as a marker and could verify all our initial results..
Cells displaying a resting membrane potential higher than −45mV and/or the series resistance was too high (> 90MΩ) were excluded from the analysis in line with standards in the field. This information is listed in the methods section lines 612ff.

Computational simulations:
All individual synaptic sections and all KCs were systematically tested in the data for figures 3 and 4. This information is listed in the figure legends. When different parameters were tested, 1000 independent trials were performed for each experiment. The information is listed in the legend of figure 5 and within the methods section.

Statistical reporting

- Statistical analysis methods should be described and justified
- Raw data should be presented in figures whenever informative to do so (typically when N per group is less than 10)
- For each experiment, you should identify the statistical tests used, exact values of N, definitions of center, methods of multiple test correction, and dispersion and precision measures (e.g., mean, median, SD, SEM, confidence intervals; and, for the major substantive results, a measure of effect size (e.g., Pearson's r, Cohen's d)
- Report exact p-values wherever possible alongside the summary statistics and 95% confidence intervals. These should be reported for all key questions and not only when the p-value is less than 0.05.

Please outline where this information can be found within the submission (e.g., sections or figure legends), or explain why this information doesn't apply to your submission:

A description of the statistical analysis is provided in the methods section (lines 694ff). The precise statistical analysis methods including exact values of N and all definitions are always listed in the corresponding figure legends.

Electrophysiology:
Raw data is shown in Figure 1 E and F, Figure 1 Supplement 1, Figure 1 Supplement 2 and in Table 1 and Table Supplement 1. The raw traces corresponding to Figure 1G that were used for the fitting of the computational model are also included in the computational dataset available at: https://doi.org/10.7281/T1/HRK27V

(For large datasets, or papers with a very large number of statistical tests, you may upload a single table file with tests, Ns, etc., with reference to sections in the manuscript.)

Group allocation

- Indicate how samples were allocated into experimental groups (in the case of clinical studies, please specify allocation to treatment method); if randomization was used, please also state if restricted randomization was applied
- Indicate if masking was used during group allocation, data collection and/or data analysis

Please outline where this information can be found within the submission (e.g., sections or figure legends), or explain why this information doesn't apply to your submission:

n/a

Additional data files ("source data")

- We encourage you to upload relevant additional data files, such as numerical data that are represented as a graph in a figure, or as a summary table
- Where provided, these should be in the most useful format, and they can be uploaded as "Source data" files linked to a main figure or table
- Include model definition files including the full list of parameters used
- Include code used for data analysis (e.g., R, MatLab)
- Avoid stating that data files are "available upon request"

Please indicate the figures or tables for which source data files have been provided:

Computational simulations:
All code and data files needed to replicate the simulations are available at:
https://doi.org/10.7281/T1/HRK27V
This includes the simulation code itself (python), the structural EM reconstruction of MBON-alpha3 (swc), the EM reconstruction of the related MBON used to model the axon and synaptic terminal structures (swc), the synapse locations as coordinate data (json), and the synapse locations by MBON section (json). Parameter values for model definition and individual simulations are specified within the code files and outlined in each figure legend where appropriate.

The data produced by each simulation is also provided (csv/dat). All files are made available as a permanent and freely accessible data collection at the Johns Hopkins University Data Archive:
https://doi.org/10.7281/T1/HRK27V
